# Early correlation of microglial activation with enhanced tumor necrosis factor-alpha and monocyte chemoattractant protein-1 expression specifically within the entorhinal cortex of triple transgenic Alzheimer's disease mice

**DOI:** 10.1186/1742-2094-2-23

**Published:** 2005-10-18

**Authors:** Michelle C Janelsins, Michael A Mastrangelo, Salvatore Oddo, Frank M LaFerla, Howard J Federoff, William J Bowers

**Affiliations:** 1 Department of Neurology, University of Rochester School of Medicine and Dentistry, Rochester, New York 14642, USA; 2 Department of Microbiology and Immunology, University of Rochester School of Medicine and Dentistry, Rochester, New York 14642, USA; 3 Center for Aging and Developmental Biology, University of Rochester School of Medicine and Dentistry, Rochester, New York 14642, USA; 4 Department of Neurobiology and Behavior, University of California, Irvine, California 92697, USA

**Keywords:** Neuroinflammation, Alzheimer's disease, beta-amyloid, pro-inflammatory molecule, microglia, 3xTg-AD, TNF-α, MCP-1

## Abstract

**Background:**

Alzheimer's disease is a complex neurodegenerative disorder characterized pathologically by a temporal and spatial progression of beta-amyloid (Aβ) deposition, neurofibrillary tangle formation, and synaptic degeneration. Inflammatory processes have been implicated in initiating and/or propagating AD-associated pathology within the brain, as inflammatory cytokine expression and other markers of inflammation are pronounced in individuals with AD pathology. The current study examines whether inflammatory processes are evident early in the disease process in the 3xTg-AD mouse model and if regional differences in inflammatory profiles exist.

**Methods:**

Coronal brain sections were used to identify Aβ in 2, 3, and 6-month 3xTg-AD and non-transgenic control mice. Quantitative real-time RT-PCR was performed on microdissected entorhinal cortex and hippocampus tissue of 2, 3, and 6-month 3xTg-AD and non-transgenic mice. Microglial/macrophage cell numbers were quantified using unbiased stereology in 3xTg-AD and non-transgenic entorhinal cortex and hippocampus containing sections.

**Results:**

We observed human Aβ deposition at 3 months in 3xTg-AD mice which is enhanced by 6 months of age. Interestingly, we observed a 14.8-fold up-regulation of TNF-α and 10.8-fold up-regulation of MCP-1 in the entorhinal cortex of 3xTg-AD mice but no change was detected over time in the hippocampus or in either region of non-transgenic mice. Additionally, this increase correlated with a specific increase in F4/80-positive microglia and macrophages in 3xTg-AD entorhinal cortex.

**Conclusion:**

Our data provide evidence for early induction of inflammatory processes in a model that develops amyloid and neurofibrillary tangle pathology. Additionally, our results link inflammatory processes within the entorhinal cortex, which represents one of the earliest AD-affected brain regions.

## Background

Alzheimer's disease (AD) is an age-related neurodegenerative disorder associated with progressive functional decline, dementia and neuronal loss. Demographics make evident that the prevalence of AD will increase substantially over the coming decades. Patients initially exhibit an inability to assimilate new information and as the disease progresses, both declarative and nondeclarative memory become ever more profoundly impaired [1]. The pervasive societal and economic burden created by this debilitating disease should provide sufficient incentive for the development of new natural history-modifying therapeutic approaches. However, because the mechanistic underpinnings of AD are incompletely understood, the clinical disease spectrum broad, and the neuropathological features of its initiation and progression limited, the development of such potential disease modifying therapies has been relatively limited.

The pathological hallmarks of the AD brain include extracellular proteinaceous deposits (plaques), composed largely of amyloid beta (Aβ) peptides, and intraneuronal neurofibrillary tangles (NFTs), which are characterized by excessive phosphorylation of tau protein. Other AD-related histopathologic features include, but are not limited to, astrogliosis, microglial activation, and reduction of synaptic integrity. These features appear to arise in a region- and time-dependent manner (reviewed in [2]). Amyloid pathology evolves in stages: early involvement is anatomically circumscribed to the basal neocortex, most often within poorly myelinated temporal areas; progression involves adjacent neocortical areas, the hippocampal formation, perforant path inclusive of its coursing through the subiculum and termination within the molecular layers of the dentate gyrus, and; finally the process involves all cortical areas [3]. Neurofibrillary tangle pathology is also progressive: Initially involving projection neurons with somata in the transentorhinal region, tangles then extend to the entorhinal region proper typically in the absence of amyloid deposition. Subsequent progression to the hippocampus and temporal proneocortex, and then association neocortex, followed by superiolateral spread and ultimately extending to primary neocortical areas [4-6]. Moreover, individuals diagnosed with mild cognitive impairment, a forme fruste of AD, display decreased entorhinal and hippocampal volume, primarily associated with diminished neuron number as compared to non-cognitively impaired controls [7-10]. These data suggest that the entorhinal cortex and hippocampus are selectively vulnerable early during the disease process.

Gaining an enhanced understanding of why these brain regions are specifically susceptible to neurodegeneration in the context of AD and elucidating the mechanisms underlying these disease processes has been the subject of intensive investigation over the past several decades. Attention has been focused upon synaptic dysfunction, due to the previously observed diminution of cholinergic synapse density and overall synapse numbers during early stages of AD [11,12]. Additionally, mouse models overexpressing human amyloid precursor protein (APP), the protein from which pathogenic Aβ peptides are proteolytically derived, exhibit decreased synaptic function antecedent to plaque deposition [13], thereby further implicating disrupted synaptic function in early stages of AD pathogenesis.

Inflammatory processes, marked by activated microglia and astrocytes in the post-mortem AD brain some of which co-localize to plaques and tangles, have long been hypothesized to contribute to AD pathogenesis [14]. The role that this response plays in the disease process, especially during pre-symptomatic stages, is not well defined. There exist multiple means by which inflammatory processes can affect neurons and potentially synaptic function in AD. Cytokines have been shown to be expressed in response to Aβ generation and a subset of these molecules have demonstrated neurotoxic activities [15-17]. Such observations imply these inflammatory molecules may serve to mechanistically link the elaboration of pathological hallmarks and synaptic dysfunction. We hypothesized that inflammation plays a role early during the disease process, at a time when synaptic dysfunction and early cognitive deficits first become evident. Disease-related inflammatory contributors to synaptic dysfunction found in early AD have long been debated, but such studies have been hampered by the lack of age-matched, early-stage human post-mortem tissue samples as well as AD-relevant animal models. In the present study, we sought to determine the temporal and region-specific expression of inflammatory molecules, previously implicated in late-stage AD, in the context of a mouse model that develops amyloid and tau pathology. A triple-transgenic model of AD (3xTg-AD) has recently been created that harbors three disease-relevant genetic alterations: a human Presenilin M146V knock-in mutation (PS1M146V), human amyloid precursor protein Swedish mutation (APPswe), and the human tauP301L mutation. These mice develop plaques and tangles in a spatial and time-dependent manner similar to pathological hallmarks observed in the brains of AD-afflicted individuals [18,19]. Most notably, this is the first animal model developed to date which facilitates the study of inflammation in the context of both amyloid and tau pathology. We performed region-specific quantitative transcript analyses and unbiased stereological counting to correlate regional and temporal changes in inflammatory molecule expression profiles to alterations in inflammatory cell numbers and AD-related pathologies. Our findings further implicate inflammatory processes as playing a role early during the disease process, and that regional differences exist that may elucidate why particular brain regions are more susceptible to AD-related disease mechanisms.

## Materials and methods

### Strains of mice

Triple transgenic (3xTg-AD) mice were created as previously described [18,19]. Age-matched 2, 3, and 6 month-old male mice were used in all studies (n = 6 per experimental group for biochemical assays, n = 4 per experimental group for quantitative stereological studies). Age-matched male C57BL/6 mice were used as non-transgenic controls in all experiments. All animal housing and procedures were performed in compliance with guidelines established by the University Committee of Animal Resources at the University of Rochester.

### Quantitative real-time PCR analysis of pro-inflammatory molecules from brain-derived RNA

RNA was isolated from microdissected hippocampus- or entorhinal cortex-enriched tissue from 2, 3, and 6 month-old 3xTg-AD and non-transgenic mice with TRIzol solution (Invitrogen, Carlsbad, CA). RNA was treated with RQ DNAse I (Promega, Madison, WI) to selectively degrade any contaminating genomic DNA, followed by phenol:chloroform extraction and ethanol precipitation. One microgram of total RNA was reverse transcribed using Applied Biosystems High-Capacity cDNA Archive Kit. An aliquot of cDNA (100 ng) was used to assess presence of 23 inflammatory targets per mouse, and was analyzed in a standard PE7900HT quantitative PCR reaction using a Taqman Assay on Demand primer probe sets in Microfluidic cards (Applied Biosystems, Foster City, CA) and 100 μL MasterMix containing HotStart DNA polymerase (Eurogentec, Belgium). 18s RNA served as the control to which all samples were normalized (Applied Biosystems, Foster City, CA). We further analyzed the data using the ΔΔC_T _method, normalizing the 3 and 6 month-old 3xTg-AD and control mouse samples to the 2 month-old 3xTg-AD and non-transgenic samples, respectively.

### Quantitative histochemical analysis of macrophages and microglia in brains of 3xTg-AD and non-transgenic mice

Age-matched 3xTg-AD and non-transgenic mice were sacrificed and processed with 4% paraformaldehyde (PFA)/PB trans-cardiac perfusions; brains were removed and post-fixed overnight with 4% PFA/PB. Sequentially, brains were transferred to 20% sucrose in PBS overnight and then 30% sucrose where they remained until sectioning. Brains were sectioned coronally (30 μm) on a sliding microtome, and stored in cryoprotectant until used for immunohistochemistry.

Sections were washed four times for 3 min. each in PB to remove cyroprotectant. To quench endogenous peroxidase activity, sections were incubated for 25 min. with 3% H_2_O_2 _(Sigma). Sections were mounted onto slides and allowed to dry. Slides were incubated in 0.15 M PB + 0.4% Triton-X100 for 5 min. at room temperature (RT; 22°C) to permeabilize the tissue. Then slides were incubated with blocking solution containing 3% normal goat serum, 3% bovine serum albumin, and 0.4% Triton-X 100 in 0.15 M PB for 1 hr. Slides were incubated with rat monoclonal anti-F4/80 antibody (Serotec, 1:100) overnight in blocking solution. Next, slides were washed eight times for 3 min. each with 0.15 M PB prior to incubation with Vectastain biotinylated goat anti-immunoglobulin (Vector Laboratories, Burlingame, CA) for 2 hrs. at RT. Excessive secondary antibody was washed in 0.15 M PB and incubated with A and B reagents (Vector Laboratories, Burlingame, CA) to conjugate HRP. Slides were developed using a DAB peroxidase kit, according to manufacturer's instructions for nickel enhancement (Vector Laboratories, Burlingame, CA).

Positively stained F4/80-expressing cells were visualized using an Olympus AX-70 microscope equipped with a motorized stage (Olympus, Melville, NY) and the MCID 6.0 Elite Imaging Software (Imaging Research, Inc.). Sections were tiled under 4× magnification. Five equal sections of entorhinal cortex and seven equal sections of hippocampus from each mouse (4 mice total) per timepoint were analyzed. Fifty percent of the defined region of interest in the entorhinal cortex or hippocampus was assessed, under 60× magnification. The coordinates from which sections were chosen for the entorhinal cortex were 2.92 mm to 4.04 mm posterior from Bregma. The sections counted in the hippocampus were from 1.70 mm to 3.40 mm posterior from Bregma.

### Qualitative immunohistochemical analysis of amyloid deposition in 3xTg-AD and non-transgenic mice

Sections were washed three times for 5 min. each, then twice for 30 min. in PBS to remove cryoprotectant. To quench endogenous peroxidase activity, sections were incubated with 3% H_2_O_2 _and 3% methanol for 25 min. Sections were then washed twice for 5 min. each with PBS, followed by epitope retrieval treatment with 90% formic acid for 5 min. at RT. Next, sections were washed twice for 5 min. each with PBS. Tissue was permeabilized with PBS + 0.1% PBS/Triton-X 100. Sections were then incubated for 1 hr at RT with PBS + 0.1% PBS/Triton-X 100 + 10% normal goat serum. Sections were incubated overnight at 4°C with primary 6E10 antibody (Signet, 1:1000) in PBS 0.1% PBS/Triton-X 100 + 1% normal goat serum. Samples were washed twice for 10 min. each with PBS + 0.1% Triton-X 100 + 1% normal goat serum prior to addition of secondary antibody. The mouse HRP ABC kit was used according to manufacturer's protocol (Vector Laboratories, Burlingame, CA). Excessive secondary antibody was washed in PBS and developed using a DAB peroxidase kit, according to manufacturer's instructions for nickel enhancement (Vector Laboratories, Burlingame, CA) and mounted on slides.

## Results

### Temporal progression of intracellular Aβ accumulation in 3xTg-AD mice

**Figure 1:**
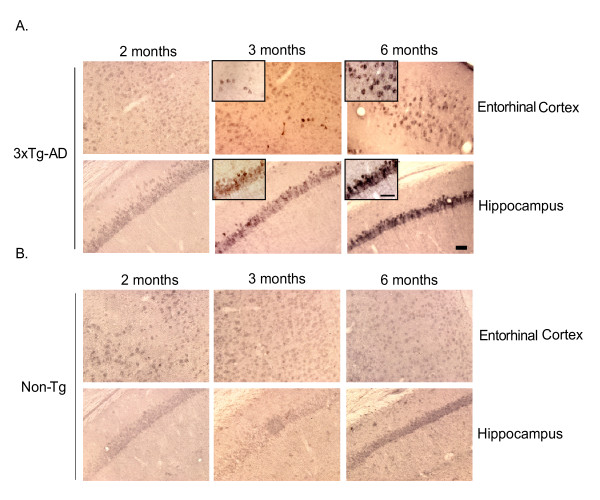
**Intracellular Aβ appears at 3 months and is enhanced by 6 months in 3xTg-AD mice**. Coronal brain sections from 2, 3 and 6 month-old 3xTg-AD and non-transgenic mice were stained with human APP/Aβ-specific 6E10 antibody and developed using DAB. Panel A illustrates that the brains of 2 month-old 3xTg-AD mice are pre-pathologic, while at 3 months, hAPP/Aβ can be readily detected in both the entorhinal cortex and hippocampus of 3xTg-AD mice. By 6 months of age, 3xTg-AD mice exhibit further enhanced deposition of hAβ in both regions. Panel B identifies sections of entorhinal cortex and hippocampus from non-transgenic mice, which are not immunohistochemically positive for endogenous mouse Aβ, therefore indicating that the 6E10 antibody specifically detects transgene-driven expression of hAPP/Aβ in 3xTg-AD mice. The scale bars depict 50 μm. The insets represent 60× magnification.

Inflammatory processes have been intimately associated with classic AD pathology in the post-mortem human brain, where evidence of astrogliosis and activated microglia in the vicinity of amyloid plaques has been readily observed [20]. Implication of inflammatory mediators in early pathogenic events during pre-symptomatic stages of AD, however, has not been clearly defined at present due to limited availability of early-stage human clinical samples and a lack of animal models that faithfully recapitulate the human disorder. The recently characterized triple-transgenic AD mouse (3xTg-AD) presently represents the most advanced animal model available in that it harbors three AD-relevant genetic alterations, which result in spatial distribution and progression of amyloid and tau pathologies strikingly similar to human AD [18,19]. To clarify the role of inflammatory processes early during disease progression, we initially assessed the age-dependent accretion of human Aβ in the entorhinal cortex and hippocampus of 3xTg-AD mice, as many posit accumulating Aβ acts as a likely early trigger of AD-related inflammatory processes [21]. The entorhinal cortex and hippocampus were the regions chosen because of the abundance of evidence implicating these regions in the earliest stages of disease [6,7,10,22]. Coronal sections from 2, 3, and 6-month old 3xTg-AD and non-transgenic mice were immunohistochemically stained with 6E10 antibody to assess extent of intracellular and extracellular human Aβ deposition. Immunohistochemical analyses revealed intracellular human Aβ staining at the 3-month time-point whereas Aβ is not detectable at 2 months of age. The number of Aβ immuno-positive cells increased by 6 months of age and the intensity of individual cell staining was significantly enhanced in 3xTg-AD mice (Fig. 1A). Extracellular accumulations or amyloid plaque-like deposits were not observed at any of these early ages. Non-transgenic mice did not exhibit Aβ staining, further confirming that the 6E10 antibody was specific for human Aβ in 3xTg-AD mice (Fig. 1B).

### Pro-inflammatory transcript profiling of 3xTg-AD and non-transgenic mouse entorhinal cortex and hippocampus reveals temporal and spatial expression of TNF-α and MCP-1

We predicted that if inflammation was involved at the earliest stages of the disease process, we would observe the coordinate expression of immunomodulatory molecules between 3 and 6 months of age, when intracellular Aβ begins to accumulate in the entorhinal cortex and hippocampus of 3xTg-AD mice. To this end, we selected a set of target inflammatory molecules that have been implicated in inflammatory responses within the central nervous system, including cytokines, chemokines, cell adhesion molecules, T cell markers and immune-related enzymes (Table 1). Many of these markers have been implicated in late-stage AD and possess the potential to influence early pathogenic processes within the regions affected early in AD. Quantitative real-time RT-PCR was performed to determine levels of these targets in microdissected entorhinal cortex and hippocampus tissue of 2, 3, and 6 month-old 3xTg-AD mice. Age-matched non-transgenic mouse samples derived from identical regions were employed as controls (n = 6 per genotype per time point). Surprisingly, we detected a 14.8-fold up-regulation of TNF-α, a pro-inflammatory modulator, and 10.8-fold increase of the chemokine MCP-1 mRNA in the entorhinal cortex of 6 month-old 3xTg-AD mice versus the 2 month-old animals (Table 2). Levels of both pro-inflammatory molecules are also slightly elevated in the 3-month 3xTg-AD entorhinal cortex, although not reaching statistical significance as compared to 2 month-old counterparts. This trending increase of TNF-α and MCP-1 transcript levels at 3 months of age correlates with the initial appearance of human transgene-derived Aβ in 3xTg-AD mice. Conversely, no detectable changes were observed in any of the assessed transcriptional targets in cDNA pools generated from hippocampal RNA samples at any of the time-points (Table 3), even though intracellular human Aβ was readily detectable within this brain region (Fig. 1A). It is remarkable that the TNF-α and MCP-1 transcript response is specific to cells resident to the entorhinal cortex, suggesting that aspects of the cellular environment may be responsible for differential inflammatory outcomes in these two disease-affected brain regions.

**Table 1:** Proinflammatory markers investigated in the temporal and spatial progression of early AD pathogenesis. Immune cell molecules/inflammatory markers were assessed from RNA isolated from entorhinal cortex and hippocampus tissue of 2, 3, and 6 month-old 3xTg-AD and non-transgenic mice by qRT-PCR using Applied Biosystems Microfluidic Cards.

**Immune Marker**	**Major Functions**
C3	complement protein, binds to pathogenic structures
CCL2 (MCP-1)	chemokine, promotes extravasation, activates macrophages, promotes Th2 immunity
CCL3	chemokine, promotes extravasation, antiviral defense, promotes Th1 immunity
Fractalkine	chemokine, involved in brain inflammation, endothelial adhesion
IP10	chemokine, antiangiogenic, promotes Th1 immmunity
TNF-α	cytokine, proinflammatory, attracts innate immune cells, activates macrophages
TGF-β	cytokine, inhibits cell growth
IL-2	cytokine, T cell growth factor
IL-6	cytokine, B and T cell growth and differentiation
IL-8	cytokine, secreted by macrophage(predominately in response to bacterial infection), recruits innate and adaptive immune cells
IL-1α	cytokine, macrophage and T cell activation
IL-1β	cytokine, macrophage and T cell activation
IL-12a	cytokine, activates NK cells, induces CD4 differentiation to Th1 cell
ICAM 1	intercellular adhesion molecule present on endothelial cells, binds LFA-1 and Mac-1 (Cd11b)
VCAM 1	adhesion molecule present on endothelial cells, binds VLA-4 integrin
CD4	cell surface marker for TH1 and TH2 T cells, coreceptor for MHC II
CD8	cell surface marker on cytotoxic T cells, coreceoptor for MHC I
CD80	cell surface marker, T cell/antigen presenting cell costimulation
CD86	cell surface marker, T cell/antigen presenting cell costimulation
Ptgs1	Cyclooxygenase type 1 (Cox-1)
Ptgs2	Cyclooxygenase type 2 (Cox-2)
Caspase 3	late-stage molecule involved in apoptosis

**Table 2:** TNF-α and MCP-1 mRNA levels are selectively elevated in the entorhinal cortex of 3xTg-AD mice prior to overt amyloid plaque pathology. Total RNA was purified from microdissected entorhinal cortex from 2, 3, and 6 month-old 3xTg-AD and non-transgenic control mice. cDNA was generated and subjected to Applied Biosystems Microfluidic Card analysis, a high-throughput quantitative RT-PCR technology that facilitates the simultaneous quantitation of 23 inflammation-related transcriptional targets. Of the panel of transcripts analyzed, only TNF-α and MCP-1 transcript levels were significantly enhanced by 6 months of age specifically within the entorhinal cortex of 3xTg-AD mice (n = 6/group). These cytokine transcripts were unchanged in the entorhinal cortex of non-transgenic mice at all time-points analyzed. *p < 0.0005 when compared to the 2 month timepoint. Proinflammatory transcript expression in 3xTg-AD and non-transgenic mice in the entorhinal cortex

	**3xTg-AD**		**Non-Transgenic**	
	
	**3 months**	**6 months**	**3 months**	**6 months**
**Marker**	**Fold Change (Relative to 2 months)**	**Fold Change (Relative to 2 months)**	**Fold Change (Relative to 2 months)**	**Fold Change (Relative to 2 months)**

C3	0.357 +/- 0.258	0.615 +/- 0.477	0.94 +/- 0.622	4.432 +/- 9.424
**MCP-1 (CCL2)**	5.079 +/- 4.826	10.796* +/- 3.298	1.459 +/- 1.230	2.616 +/- 5.184
CCL3	0.317 +/- 0.279	0.521 +/- 0.388	0.754 +/- 0.394	0.414 +/- 0.170
Fractalkine	0.853 +/- 0.215	0.858 +/- 0.333	1.584 +/- 0.568	0.989 +/- 0.311
IP10	1.291 +/- 0.911	1.922 +/- 1.028	1.157 +/- 0.507	1.393 +/- 1.062
**TNF-α**	5.299 +/- 4.580	14.822* +/- 5.618	1.215 +/- 1.793	1.702 +/- 1.916
TGF-β	1.149 +/- 0.181	1.092 +/- 0.527	1.054 +/- 0.178	0.922 +/- 0.312
IL-2	1.292 +/- 0.830	1.900 +/- 2.225	0.811 +/- 0.218	1.459 +/- 0.740
IL-6	2.216 +/- 2.921	4.900 +/- 5.817	1.632 +/- 1.604	3.340 +/- 4.726
IL-8	0.110 +/- 0.075	0.190 +/- 0.180	0.350 +/- 0.234	0.424 +/- 0.418
IL-1α	0.932 +/- 0.243	1.371 +/- 0.687	0.788 +/- 0.290	0.568 +/- 0.285
IL-1β	0.413 +/- 0.478	0.899 +/- 0.925	1.528 +/- 1.502	0.893 +/- 0.859
IL-12α	0.402 +/- 0.182	0.444 +/- 0.346	15.159 +/- 22.054	13.181 +/- 13.192
ICAM 1	1.000 +/- 0.317	1.215 +/- 0.567	1.029 +/- 0.076	0.733 +/- 0.202
VCAM 1	1.031 +/- 0.067	0.969 +/- 0.399	0.890 +/- 0.137	0.690 +/- 0.337
CD4	3.149 +/- 2.363	2.191 +/- 1.772	0.549 +/- 0.488	0.605 +/- 0.440
CD8	0.876 +/- 0.603	2.190 +/- 1.978	0.329 +/- 0.433	9.954 +/- 17.454
CD80	0.888 +/- 0.158	0.812 +/- 0.460	0.822 +/- 0.473	0.714 +/- 0.451
CD86	0.839 +/- 0.237	0.843 +/- 0.394	1.002 +/- 0.207	0.628 +/- 0.237
Ptgs1	1.066 +/- 0.225	1.270 +/- 0.609	1.165 +/- 0.232	0.966 +/- 0.323
Ptgs2	0.725 +/- 0.265	0.538 +/- 0.261	2.105 +/- 1.357	1.369 +/- 0.485
Caspase 3	0.955 +/- 0.171	0.900 +/- 0.424	0.884 +/- 0.173	0.741 +/- 0.366
VEGF	0.887 +/- 0.214	0.811 +/- 0.266	0.959 +/- 0.229	0.974 +/- 0.618

*p < 0.0005, student t test:two sample equal variance

**Table 3:** Inflammation-related transcript levels remain stable in the hippocampus of 2, 3, and 6 month-old 3xTg-AD and control mice. Total RNA was purified from microdissected hippocampus from 2, 3, and 6 month-old 3xTg-AD and non-transgenic control mice. cDNA was generated and subjected to Applied Biosystems Microfluidic Card analysis of 23 inflammation-related transcriptional targets. None of the transcriptional targets assessed exhibited altered expression at any of the assessed time-points. Proinflammatory transcript expression in 3xTg-AD and non-transgenic mice in the hippocampus

	**3xTg-AD**		**Non-Transgenic**	
	
	**3 months**	**6 months**	**3 months**	**6 months**
Marker	**Fold Change (Relative to 2 months)**	**Fold Change (Relative to 2 months)**	**Fold Change (Relative to 2 months)**	**Fold Change (Relative to 2 months)**

C3	1.181 +/- 0.855	1.006 +/- 0.473	0.754 +/- 0.385	1.123 +/- 0.734
**MCP-1 (CCL2)**	0.554 +/- 0.72	0.204 +/- 0.122	1.864 +/- 0.756	1.392 +/- 0.709
CCL3	0.621 +/- 0.353	0.571 +/- 0.339	0.664 +/- 0.305	0.638 +/- 0.369
Fractalkine	0.619 +/- 0.136	0.718 +/- 0.256	0.973 +/- 0.086	0.919 +/- 0.240
IP10	0.347 +/- 0.154	0.388 +/- 0.246	1.185 +/- 0.552	0.796 +/- 0.481
**TNF-α**	0.238 +/- 0.278	0.216 +/- 0.195	0.899 +/- 0.432	0.461 +/- 0.348
TGF-β	0.683 +/- 0.187	0.793 +/- 0.138	1.117 +/- 0.178	0.935 +/- 0.468
IL-2	0.650 +/- 0.077	0.637 +/- 0.055	0.360 +/- 0.228	0.462 +/- 0.447
IL-6	0.373 +/- 0.215	0.531 +/- 0.524	0.719 +/- 0.320	0.396 +/- 0.506
IL-8	0.180 +/- 0.228	0.198 +/- 0.170	0.292 +/- 0.202	0.206 +/- 0.105
IL-1α	0.602 +/- 0.111	0.838 +/- 0.229	0.766 +/- 0.431	0.825 +/- 0.552
IL-1β	0.449 +/- 0.687	0.517 +/- 0.885	1.489 +/- 1.711	1.672 +/- 1.570
IL-12α	1.597 +/- 2.736	3.329 +/- 7.112	0.362 +/- 0.364	0.678 +/- 0.747
ICAM 1	0.771 +/- 0.174	0.756 +/- 0.162	1.075 +/- 0.163	0.941 +/- 0.324
VCAM 1	0.730 +/- 0.175	0.738 +/- 0.173	0.943 +/- 0.191	0.833 +/- 0.337
CD4	0.603 +/- 0.751	0.681 +/- 0.822	1.258 +/- 0.750	1.190 +/- 0.847
CD8	0.534 +/- 0.484	0.943 +/- 1.047	1.127 +/- 1.011	10.462 +/- 10.77
CD80	0.464 +/- 0.353	0.349 +/- 0.249	1.116 +/- 0.496	1.117 +/- 1.079
CD86	0.614 +/- 0.126	0.614 +/- 0.163	0.893 +/- 0.112	0.686 +/- 0.307
Ptgs1	0.771 +/- 0.153	0.913 +/- 0.262	1.013 +/- 0.105	1.025 +/- 0.287
Ptgs2	0.649 +/- 0.309	0.936 +/- 0.351	1.060 +/- 0.092	0.868 +/- 0.370
Caspase 3	0.556 +/- 0.145	0.569 +/- 0.119	0.830 +/- 0.187	0.697 +/- 0.243
VEGF	0.728 +/- 0.196	0.723 +/- 0.169	0.819 +/- 0.111	0.809 +/- 0.257

### Increased microglial/macrophage numbers in the entorhinal cortex correlates with enhanced TNF-α and MCP-1 expression

**Figure 2:**
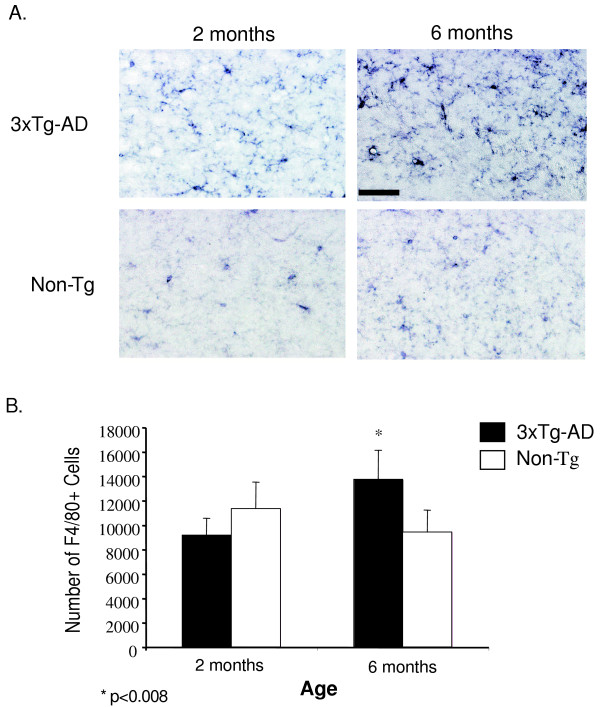
**The 3xTg-AD entorhinal cortex harbors an increased number of macrophages/microglia at 6 months of age**. Coronal brain sections from 2 and 6 month-old 3xTg-AD and control mice were stained with anti-F4/80 antibody and developed using DAB. (A) Qualitative image analysis reveals activation of F4/80-expressing macrophages and microglia specifically in the entorhinal cortex of 3xTg-AD mice at 6 months of age. (B) Unbiased quantitative stereologic analyses were performed on the entorhinal cortex to derive the total number of F4/80-positive cells. Error bars indicate standard deviation. N = 4 per genotype per time point. "*" indicates p < 0.008. The scale bar represents 50 μm.

**Figure 3:**
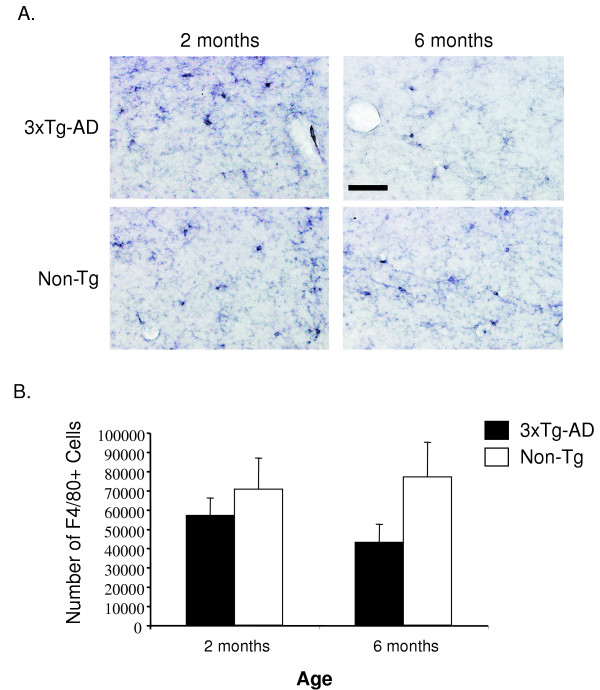
**The 3xTg-AD hippocampus does not have an increased number of macrophages/microglia at 6 months of age**. Coronal brain sections from 2 and 6 month-old 3xTg-AD and control mice were stained with the anti-F4/80 antibody and developed using DAB. (A) Qualitative analyses shows little change in activation of microglia and macrophages in the hippocampus of 3xTg-AD or non-transgenic mice over time. (B) Unbiased stereology was performed on the hippocampal formation to determine total number of F4/80-positive cells. Error bars indicate standard deviation. N = 4 per genotype per time point. The scale bar represents 50 μm.

Our finding that specific TNF-α and MCP-1 transcript expression within the entorhinal cortex of 3xTg-AD mice indicates that a regional difference exists between the entorhinal cortex and hippocampus that would elaborate a time-dependent increase in the intensity of inflammatory processes. This difference appears to be independent of solely intracellular human Aβ accumulation since transgene expression is extant in both regions at 3 and 6 months. Because microglia and macrophages represent likely candidate(s) of TNF-α and MCP-1 production [23,24], we assessed the total number of cells in the entorhinal cortex and hippocampus of transgenic and non-transgenic mice to determine if differences exist that may explain regional differences of inflammatory cytokine expression profiles. Coronal sections containing the entorhinal cortex and hippocampus of 2 and 6-month 3xTg-AD and control mice were immunohistochemically stained with anti-F4/80 antibody to identify resident microglia and macrophages. Microscopic analysis revealed that the microglial phenotype in the entorhinal cortex of 3xTg-AD mice was that of a highly activated state as shown by enhanced staining and cellular morphology at 6 months of age (Fig. 2A). Minimal differences in microglial activation state were apparent in identical regions in non-transgenic control animals (Fig. 2A). To quantify cell number in this region, we performed unbiased stereology on 3xTg-AD and control entorhinal cortex sections. We detected a significant increase in the total number of F4/80-positive cells in the entorhinal cortex of 6 month-old 3xTg-AD mice as compared to 2 month-old counterparts (Fig. 2B). There was no change in the number of F4/80-positive cells in the entorhinal cortex of control mice, indicating that the increase in microglial cell number in transgenic mice was not due to normative aging. Assessment of F4/80-positive cells in the hippocampus revealed no detectable alteration between 2 and 6 month-old 3xTg-AD and control mice in terms of microglial activation status (Fig. 3A). Quantification using unbiased stereology indicated no significant differences in F4/80-positive cell numbers between the 2 and 6-month time-points (Fig. 3B).

## Discussion

Dissecting the role that inflammation plays early in AD is challenging, as AD is a complex chronic disorder with varying pathologic sequelae from which the underlying causative mechanisms are unknown. Activation of microglia and astrocytes, and the presence of many inflammatory mediators, including cytokines, chemokines and complement proteins have been only identified in the post-mortem AD brain in the vicinity of senile plaques and NFTs [20,25]. This observation leads one to question if inflammation is involved early during the course of AD and, if so, how does it contribute to pathogenesis? Understanding the earliest events is of utmost importance, as inflammation may represent a viable therapeutic target of AD. Interestingly, retrospective studies assessing the effects of non-steroidal anti-inflammatory drugs (NSAIDs) on nondemented individuals have shown decreased risk of developing AD when these individuals utilized NSAIDs for prolonged periods of time [26,27]. Our studies aimed to identify the earliest period during which inflammatory processes initiate in the 3xTg-AD mouse model [19]. Our results illustrate 3 main points: 1) Inflammatory processes precede significant extracellular amyloid plaque deposition in the 3xTg-AD brain, substantiated by increased TNF-α and MCP-1 transcript levels, coincident temporally with the production of intracellular Aβ accumulation. 2) The expression of these molecules is spatially localized to the entorhinal cortex but not hippocampus at the early time-points assessed. 3) There is a marked increase in the number of microglia and macrophages in the entorhinal cortex that correlates with when TNF-α and MCP-1 transcript levels are significantly up-regulated.

In the late-stage AD brain, it has been shown that inflammatory molecules are produced primarily by microglia and astrocytes as they respond to plaques and neuronal damage [17]. Our finding of increased TNF-α and MCP-1 expression prior to significant plaque deposition in 3xTg-AD mice, which occurs extensively at 12 months [19], may represent a contributory role between inflammatory processes and early AD pathogenesis. Precisely how these molecules impart effects in 3xTg-AD mice at this early time-point is not certain; however, recent evidence has suggested that TNF-α and MCP-1, as well as other pro-inflammatory molecules may play a role in inhibiting microglial phagocytosis of fibrillar Aβ *in vitro *[28]. Likewise, increased inflammatory responses and subsequent secretion of cytokines, in particular, IL-1β, may play an important role in tau phosphorylation in the 3xTg-AD model [29]. In this study, activation of microglia by LPS only affected the tau pathology via cdk5/p25 activation, but not the amyloid pathology, further highlighting the potential pathophysiological changes that can be induced by inflammation in AD. Certainly, inflammatory mediators have been implicated as being both protective and exacerbating, depending on the model system and the levels of cytokine present [30].

TNF-α can be expressed by astrocytes, microglia and neurons in response to various stimuli in the CNS [17]. Initially, TNF-α is an innate mediator, promoting chemokine and cytokine expression and extravasation of other immune cells. One possible mechanism that may implicate TNF-α in contributing to AD pathogenesis is evidence that it can increase Aβ peptide production [31]. Additionally, inflammatory molecule signaling may cause increased cleavage of APP by the γ-secretase complex, whereby TNF-α, IL-1β, and IFN-γ have been shown to enhance production of Aβ peptides via a γ-secretase-dependent mechanism *in vitro*. Moreover, antagonizing TNFR1 signaling can lead to diminished γ-secretase activity [32]. Further evidence supporting pathogenic effects of TNF-α-mediated signaling is TNFR1 and TRADD, a TNF receptor adaptor protein that allows for NF-κB and JNK activation, are both increased in AD tissue and APPswe mice. This increase is correlative with TUNEL-positive neurons in primary hippocampal cultures [33]. Collectively, these observations suggest TNF-α contributes to aberrant APP processing and initiation of pro-apoptotic pathways.

MCP-1 is a chemokine that is expressed by microglia and astrocytes that facilitates extravasation of immune cells expressing its cognate receptor, CCR2, to cross the blood brain barrier and guides them to the site of damage. As with TNF-α, the role of MCP-1 in AD pathophysiology is uncertain. A recent study of APPswe/CCL2 (MCP-1) bigenic mice showed increased diffuse Aβ deposition, as compared to APPswe mice at 14 months of age. Since changes were not observed in APP processing, the authors concluded that MCP-1 overexpression in APPswe mice correlated with diminished clearance of Aβ [34]. Overall, it is interesting that of the 23 immunomodulatory markers assessed in our study, TNF-α and MCP-1 were the only two that changed significantly over time, possibly signifying their importance during nascent stages of AD pathogenesis. Perhaps, the other inflammatory targets are triggered at later stages of the disease in response to further neurodegenerative events.

Exogenously applied Aβ can trigger the expression of cytokines *in vitro *and when injected directly into the mouse brain [24,35]. However, the fascinating result in our study is that expression of TNF-α and MCP-1 was detected specifically within the entorhinal cortex and not the hippocampus, despite the fact that immunocytochemically detectable intraneuronal Aβ increased over time in both brain regions. Additionally, although not statistically significant, TNF-α and MCP-1 transcript levels were elevated at 3 months of age in 3xTg-AD mouse entorhinal cortex, and were increased to statistical significance by 6 months of age suggesting a state of chronic up-regulation and positive-feedback for the expression of both of these inflammatory molecules. Therefore, we are unable to conclude, as detected by the methodology employed in this study, that intracellular Aβ accumulation is the sole contributing factor promoting TNF-α and MCP-1 transcript expression specifically in the entorhinal cortex. It is possible that these molecules are neuronally expressed within the entorhinal cortex because they are inherently more sensitive to accumulating Aβ, as other neurons were shown previously to express both of these molecules during times of stress [17]. Another possibility is that the entorhinal cortex elaborates inflammatory molecule expression owing to the structure of Aβ elaborated. For example, oligomeric Aβ is posited to be the more toxic structural intermediate arising during Aβ fibrillogenesis, and this form has been shown to readily induce cytokine expression *in vitro *[36]. Regional differences in intracellular and extracellular oligomeric Aβ profiles *in vivo *may, therefore, account for the regional specificity of cytokine/chemokine expression and microglial activation that we observed in the 3xTg-AD mice. Conversely, the regional elaboration of TNF-α and MCP-1 may occur via an Aβ-independent mechanism and caused by an environmental stimulus, such as region-selective oxidative stress. Practico and colleagues demonstrated that lipid peroxidation in the APPswe brain can occur prior to Aβ deposition in APPswe mice [37], suggesting that disruption of cellular membrane homeostasis could also contribute to inflammatory molecule induction, and perhaps, regional differences in lipid peroxidation profiles are responsible for our region-specific observations in 3xTg-AD mice.

The significant increase in the number of microglia and macrophages in the entorhinal cortex from 2 to 6 months of age in 3xTg-AD mice is coincident with the increase of TNF-α and MCP-1. We believe this could be due to microglial proliferation, activation of the resting resident population of brain microglia and macrophages and/or recruitment of peripheral macrophage-like cells (F4/80-positive) from outside the brain. Macrophages express CCR2 and thus, are capable of responding to a compromised entorhinal cortex via chemotaxis. Whether the observed increase in F4/80^+ ^cell number indicates a homeostatic or pathologic response is not clear. APPswe/CCL2 mice demonstrate enhanced microglial numbers that are concurrent with increased extracellular Aβ deposition, that the authors postulate is due to an inability to effectively clear Aβ [34]. This may relate partially to the increased ApoE levels observed in APPswe/CCL2 mice produced by microglia and macrophages. If a similar mechanism is at play in the 3xTg-AD mouse model, this finding suggests a pathogenic role for these cells in initiating degeneration within the entorhinal cortex.

In summary, our results indicate a potential early role for inflammatory processes in the temporal and spatial evolution of AD pathogenesis. Because TNF-α and MCP-1 are produced specifically within the entorhinal cortex where human AD has been shown to arise, these molecules are likely playing an instrumental role in disease perpetuation. This work provides insight into the involvement of TNF-α and MCP-1 mediated inflammation in the temporal and spatial progression of early AD pathogenic events and may potentially herald new therapeutic targets. Our use of the 3xTg-AD model to assess these early events is unique, as all previous studies have examined inflammatory processes in the context of either amyloid or tau pathology, but not both. This transgenic mouse allows us to directly examine the dynamic interplay of inflammation, Aβ pathology, and tau dysfunction. Modulating TNF-α and MCP-1 function in future studies will elucidate how these inflammatory mediators influence the severity and progression of AD-related pathology and synaptic dysfunction.

## List of abbreviations

AD: Alzheimer's disease

APP: Amyloid precursor protein

APPswe: Amyloid precursor protein Swedish mutation

PS1: Presenilin 1

Aβ : Beta-amyloid

TNF-α : Tumor necrosis factor-alpha

MCP-1: monocyte chemoattractant protein-1

Tg: Transgenic

PBS: Phosphate-buffered saline

## Competing interests

The author(s) declare that they have no competing interests.

## Authors' contributions

MCJ carried out the quantitative real-time PCR, 6E10 immunohistochemistry, F4/80 immunohistochemistry, stereology, experimental analysis and data interpretation, and prepared the manuscript. MAM performed tissue microdissection, brain sectioning, and 6E10 immunohistochemistry. SO and FML conceived the design of and generated the 3xTg-AD mouse model. HJF and WJB conceived the design of the study, aided in the preparation of the manuscript, and provided critical analysis of the manuscript.
